# Inkjet printing and UV-LED curing of photochromic dyes for functional and smart textile applications†

**DOI:** 10.1039/c8ra05856c

**Published:** 2018-08-08

**Authors:** Sina Seipel, Junchun Yu, Aravin P. Periyasamy, Martina Viková, Michal Vik, Vincent A. Nierstrasz

**Affiliations:** Textile Materials Technology, Department of Textile Technology, Faculty of Textiles, Engineering and Business, University of Borås 501 90 Borås Sweden sina.seipel@hb.se; Department of Material Engineering, Faculty of Textile Engineering, Technical University of Liberec 461 17 Liberec Czech Republic

## Abstract

Health concerns as a result of harmful UV-rays drive the development of UV-sensors of different kinds. In this research, a UV-responsive smart textile is produced by inkjet printing and UV-LED curing of a specifically designed photochromic ink on PET fabric. This paper focuses on tuning and characterizing the colour performance of a photochromic dye embedded in a UV-curable ink resin. The influence of industrial fabrication parameters on the crosslinking density of the UV-resin and hence on the colour kinetics is investigated. A lower crosslinking density of the UV-resin increases the kinetic switching speed of the photochromic dye molecules upon isomerization. By introducing an extended kinetic model, which defines rate constants *k*_colouration_, *k*_decay_ and *k*_decolouration_, the colour performance of photochromic textiles can be predicted. Fabrication parameters present a flexible and fast alternative to polymer conjugation to control kinetics of photochromic dyes in a resin. In particular, industrial fabrication parameters during printing and curing of the photochromic ink are used to set the colour yield, colouration/decolouration rates and the durability, which are important characteristics towards the development of a UV-sensor for smart textile applications.

## Abstract 2

Tuned performance of an inkjet-printed and UV-LED cured smart textile UV-sensor based on a photochromic dye using fabrication parameters.

## Graphical abstract

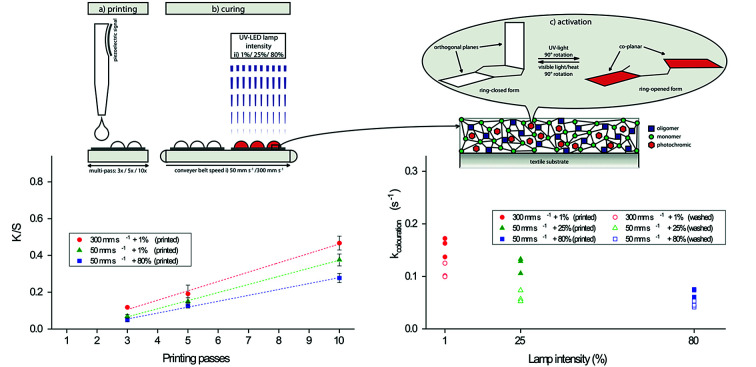

## Introduction

The main drivers and motivation behind the sensor technology of smart textiles are the changing environment, an increasing need for protection mechanisms and a demand for lightweight products with highly integrated functions. Smart textiles help the user to sense and respond to external stimuli, and ideally adapt to them.^1^ The need for UV-sensors to detect UV rays from sunlight is demonstrated using a variety of materials like hydrated tungsten oxide nanosheets,^2^ conductive polymer and CNT composites,^3^ fluorescent polyoxometalate and viologen,^4^ multifunctional ZnO-based biofilms^5^ and various photochromic compounds^6^ in recent research studies. For smart textile applications, photochromic materials provide excellent properties to function as flexible sensors, which display a reversible colour change and therewith warn the user of the presence of harmful UV-radiation in everyday situations where UV-light is not an obvious threat, *i.e.* windy and partially cloudy weather conditions.

Upon UV-irradiation photochromic dyes such as organic spiro-compounds reversibly change colour, from colourless to a colour. Elevated photoelectric energy rearranges the molecular structure of the dye, which is reversible either through visible light or heat. The isomers A and B of the dye have different absorption spectra.^7^

In recent literature, the development of UV-sensing textiles using photochromic dyes has been paid much attention. The integration of photochromic dyes into textile structures has been proven successful with traditional textile production techniques such as screen-printing,^8–10^ dyeing^11,12^ and mass dyeing.^10,13,14^ Moreover, the application of photochromic dyes using novel production techniques has emerged. Whereas, Aldib^15^ investigated the potential of digital inkjet printing of solvent-based ink to produce photochromic textiles, Fu *et al.*^16^ studied photonic curing to produce a photochromic cross-linked polymer. In other areas of textile colouration such as pigment printing, the advantages of inkjet printing in combination with photo-curing of an aqueous polyurethane acrylate system^17^ or by mini-emulsion encapsulation^18^ were explored. To the best of our knowledge, the combination of digital inkjet printing and UV-LED curing has not yet been studied in the industrially applicable fabrication of a UV-sensing smart textile using photochromic dye.

**Fig. 1:**
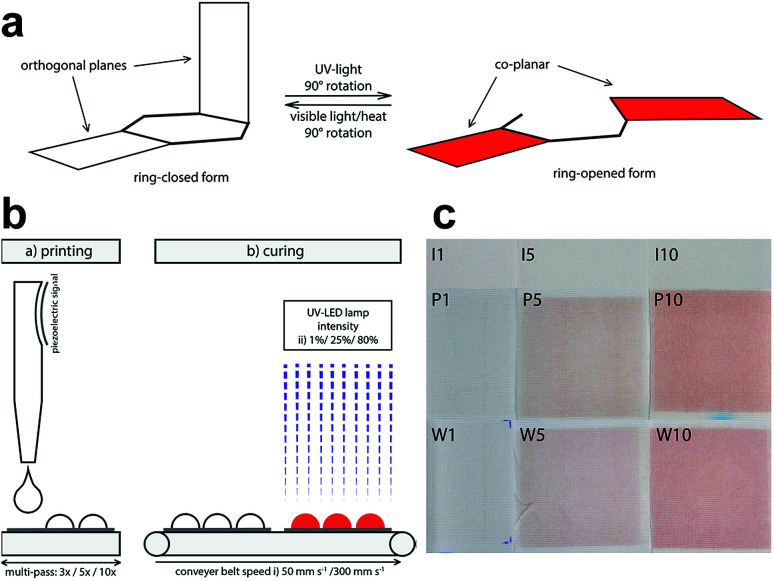
(a) Isomerization of a photochromic dye between its colourless ring-closed and coloured ring-opened form. The planes indicate the remaining chemical groups of the heterocyclic organic compound that partially undergo a 90° rotation followed by cleavage of the spiro-carbon-oxygen. (b) Production process scheme of photochromic prints. (1) Printing of various passes of the UV-curable photochromic ink on PET. (2) Substrate transportation with a belt speed of 50 mm s^−1^ or 300 mm s^−1^ and curing with 1, 25 or 80% of the maximum UV-LED lamp power. (c) Inactive (I) and active printed (P) and washed (W) photochromic prints with 1, 5 and 10 printing passes cured at 50 mm s^−1^ and 80%.

UV-curable coatings distinguish themselves from thermally dried coating or binder systems, such as water-based acrylate binders typically used in screen-printing, by their high rigidity and three-dimensional cross-linked structure.^19^ According to Schwalm *et al.*^20^ and Agarwal,^21^ depending on the chemistry, *i.e.* the choice of oligomers and monomers in the formulation, the coating's flexibility, adhesion and hardness can be controlled. Substrate-ink adhesion and flexibility are particularly important when printing on flexible substrates like textiles. In general, UV-curable inkjet inks exhibit good adhesion to porous substrates as compared to solvent-based alternatives.^22^ Inkjet printing and UV-curing also provide a controllable process, where the thickness of the printed ink and the cross-linked network can be controlled. Eventually, a high degree of crosslinking is desired to achieve good adhesion of dyes in a solid ink matrix onto the textile surface and to ensure a durable functional surface treatment. The degree of polymer crosslinking of the carrying ink also affects the rate of the switching reaction of photochromic dyes such as spirooxazines or naphthopyrans between their ring-closed colourless and ring-opened coloured state (Fig. 1(a)). A 90° rotation in the molecular structure between non-planar and planar isomers requires space.^7,23,24^ Hence, the structural molecular changes of the dyes upon isomerization are largely affected by the properties of the host matrix. Whereas, Ercole *et al.*^25^ and Malic *et al.*^26^ studied the placement of naphthopyran dye in host polymers, Mutoh *et al.*^27^ investigated block copolymers as host matrix. Other factors, which influence the photochromic response are chain length, free volume and rigidity of the host matrix.^24,27,28^ In particular, the higher the crosslinking density of the ink, the lower the degree of freedom in the matrix and hence the slower the switching reaction of the photochromic dye is.^25,26,29^

In this paper, we explore and tune the colour performance of a novel UV-responsive textile using fabrication parameters of an industrially applicable process. Altering the curing intensity of the photochromic ink on the textile during fabrication, influences the rigidity of the UV-resin, the dye-polymer matrix interaction and hence the degree of freedom of the dye to switch between isomers in the matrix. In particular, the photo-switching of the photochromic textiles is influenced by the combination of two challenging functions of electromagnetic radiation during production and characterization. The photochromic textile, which is fabricated by inkjet printing and UV-LED curing is activated by UV-light once in use. The impact of UV-rays during curing and activation requires the introduction of an extended kinetic model to describe the kinetics of UV-curable photochromic materials. The lowest curing intensity achieves highest colour yields and fastest switching behaviour between the coloured and colourless state of the photochromic dye Ruby Red in a UV-curable resin. The proposed kinetic model is an important step towards the development of a smart textile UV-sensor and allows the prediction of the colour performance based on fabrication parameters.

## Materials and methods

### Materials

A commercial heterocyclic spiro-compound (Reversacol Ruby Red) from Vivimed Labs, UK was used as photochromic dye in a UV-curable inkjet ink formulation to produce a textile UV-sensor. The photochromic ink contains a dye concentration of 2.5 g L^−1^. The UV-curable carrier consists of dipropylene glycole diacrylate monomers (DPGDA), amine modified polyetheracrylate oligomers (Ebecryl 81) supplied by Allnex SA/NV, Belgium and a UV-LED photo-initiator (Genocure TPO-L) supplied by Rahn AG, Switzerland. Ethyl acetate, 99.9% (Chromasolv Plus), purchased from Sigma-Aldrich was used as media to disperse the photochromic dye in the ink formulation, but was removed afterwards by vacuum pumping. Plain-woven polyester (PET) fabric with a weight of 147 g m^−2^ and 20 and 23 threads cm^−1^ in warp and weft direction, respectively, received from FOV Fabrics, Sweden was used as the substrate for digital inkjet printing.

### Production of photochromic prints

Photochromic prints were produced using a Sapphire QS 10 print head from Fujifilm Dimatix, USA. Printing was carried out in multi-pass mode with a resolution of 300 dpi and at a print head temperature of 35 °C. To determine the effect of the amount of ink deposition on the crosslinking density and colour behaviour, different amounts of ink were deposited during printing. 1, 3, 5, 7 and 10 passes of ink were printed on PET, which correspond to ink amounts of 3, 8, 10, 13 and 19 g m^−2^, respectively. Subsequently, the prints were cured using a UV-LED lamp FireJet from Phoseon Technology, USA with emission wavelengths of 380 to 420 nm and a maximum emission power of 6 W cm^−2^ (the UV-LED lamp is part of a continuous pilot-scale inkjet printing system with a conveyer belt for substrate transportation). To determine the optimal curing efficiency and colour performance, curing conditions were varied. Hence, two different conveyer belt speeds, 50 mm s^−1^ and 300 mm s^−1^, and three different lamp intensities, 1%, 25% and 80%, were used (Fig. 1(b)). To determine the effect of a cleaning step, one set of samples was washed once after printing and curing. Washing was done using procedure 4 N and reference detergent 3 according to ISO 6330:2012. The samples were dried using drip flat drying.

### UV-Vis spectroscopy

UV-Vis spectra were collected using a Libra S60 double beam spectrophotometer from Biochrom, UK and a UV-3101PC from Shimadzu, Japan double beam spectrophotometer with integrated sphere accessory to affirm fit of the photo-initiator for the UV-LED lamp of the pilot-scale inkjet printing system and to determine the influence of UV-varnish on the absorption spectra of the dye.

### Colour analysis

**Fig. 2:**
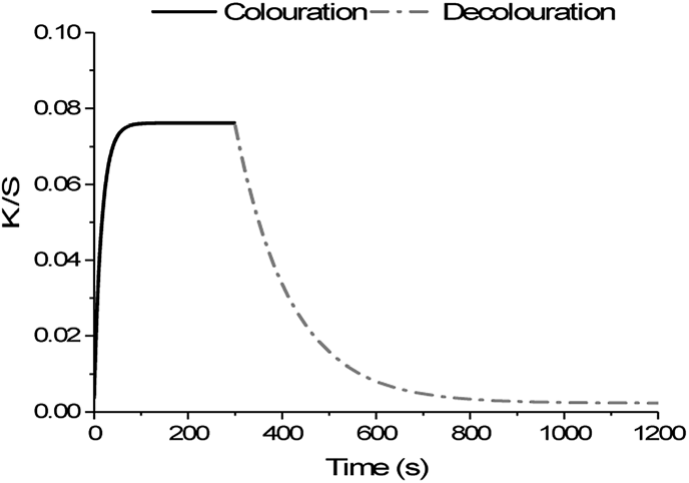
Scheme of colour measurement cycle with 300 s of UV-exposure for colouration and 900 s of relaxation for decolouration.

The colour behaviour of the photochromic prints was measured using an LCAM Photochrom 3 spectrophotometer, which allows continuous colour measurement upon cycles of UV-exposure and relaxation^30^ (Fig. 2).

The activation light source was an Edixon UV-LED lamp (EDEV-3LA1) with a radiometric power *Φ*_V_ of 350 mW and an emission peak with wavelengths between 395 nm and 410 nm. The measuring light source was a dual light source system of combined high power white LEDs with CCTs of 4000, 5000 and 7000 K. The measuring light source system illuminates samples with illuminance of 60 klx. This illuminance is a compromise between brightest sunlight intensity (120 klx) and daylight intensity without direct sunlight at noon (20 klx). In addition, the measuring light source is equipped with a set of high-pass filters preserving light transmission bellow 420 nm, which decrease possible activation of photochromic composition. Colour values *K*/*S* were measured from reflectance values *R* using the Kubelka–Munk function:1
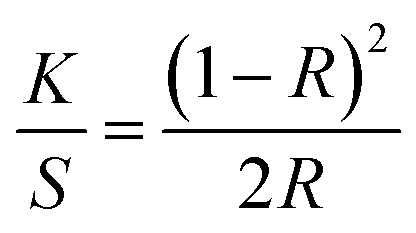


The kinetic model shown in eqn (2) follows first-order kinetics and is generally used to describe photochromic colour behaviour for both the colouration and decolouration reaction as seen in Fig. 2.^31^2
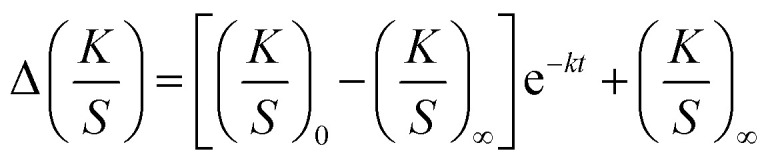


Based on the kinetic model, the sensor functionality was specified by its achieved colour intensity Δ*K*/*S* upon activation with UV-light, its rate constant of colour increase *k*_colouration_ to achieve maximum colouration *K*/*S*_∞_, and its rate constant of colour decrease *k*_decolouration_ to revert to the initial colourless state *K*/*S*_0_.

### DSC analysis

The influence of deposited ink amount and curing conditions on the curing efficiency was determined indirectly by analysing the shift of the melting peak *T*_m_ of PET using a Q1000 DSC from TA Instruments, USA. The DSC samples sealed in aluminum pans were weighted. The balance (Kern) has 99.96% accuracy when calibrated against a standard weight of 100 mg. The heat flow of samples of about 5.0 mg was measured during a heating and cooling cycle from 25 °C to 290 °C and back to 25 °C with a rate of 10 °C min^−1^. The released energy Δ*H* of the endothermic reaction during melting of the material gives indirect information on the degree of polymer crosslinking at the interface between the PET fabric and the photochromic UV-cured ink. The shift of the melting peak *T*_m_ towards higher temperatures compared to the *T*_m_ of pure PET fabric indicates that an insulating layer is built surrounding the textile yarn/fiber. Depending on the curing intensity, the insulation effect of the print varies. The adhesion of the ink on the PET surface is indicated by the difference in melting peak temperature Δ*T*_washed_ of the photochromic prints before and after washing. Washing hence gives information about the durability of the photochromic, UV-cured surface treatment on PET.

### Statistical analysis

Statistical analysis of colour measurement and DSC data was conducted in Origin 2017 from OriginLab Corporation, USA at a confidence interval of 95%. One-way ANOVAs were used to determine the effect of washing on the crosslinking density of the ink, colour yields and rate constants of the dye. Two-way ANOVAs were used to determine the effect of the production parameters belt speed and lamp intensity, as well as the significance of their interaction.

## Results and discussion

### Design of the UV-curable ink

UV-Vis spectra confirm the fit of photo-initiator (absorption peak between 340 and 410 nm) used in the ink formulation with the UV-LED light source (emission band of 380 to 420 nm) to initiate polymer crosslinking. The normalized absorption peaks of the photochromic dye in solvent and UV-resin conform to each other. Ruby Red has an absorption peak of 500 nm in ethyl acetate and 490 nm in the UV-curable resin (Fig. S1 in ESI†). Analysis of the measured colour values, rate constants for colour development and reversion are hence made at a wavelength of 500 nm.

### DSC analysis of crosslinks in the UV-curable ink

#### Effect of deposited ink amount and curing settings on the crosslinking density

**Fig. 3:**
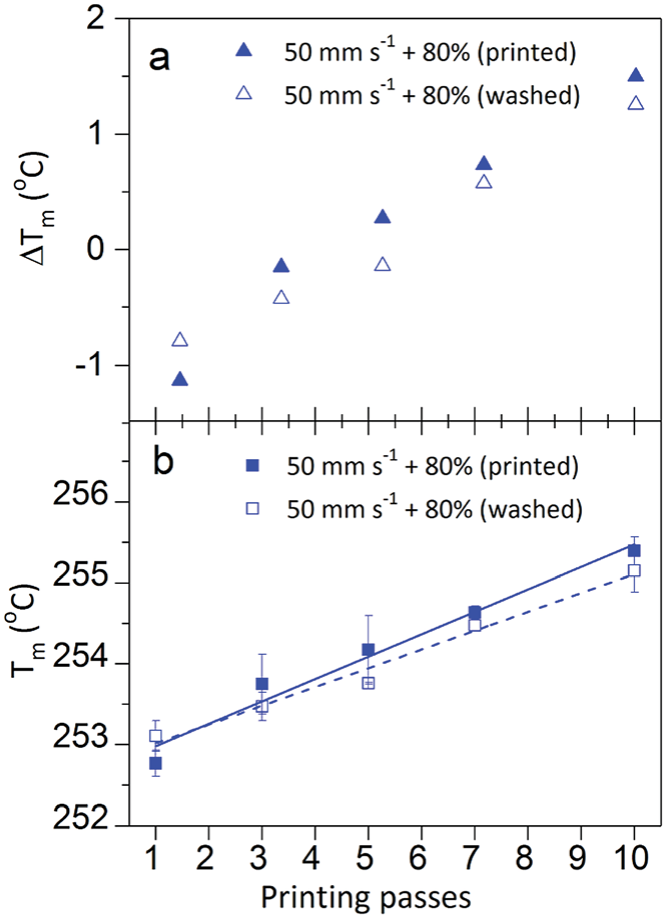
(a) Melting peak temperature difference Δ*T*_m_ between UV-cured prints and non-printed PET, after printing (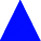) and washed (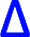). The prints are cured at 50 mm s^−1^ belt speed and 80% lamp intensity (b) linear increase of *T*_m_ for 1- to 10-pass prints cured at 50 mm s^−1^ belt speed and 80% lamp intensity printed (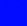) and washed (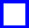).

**Fig. 4:**
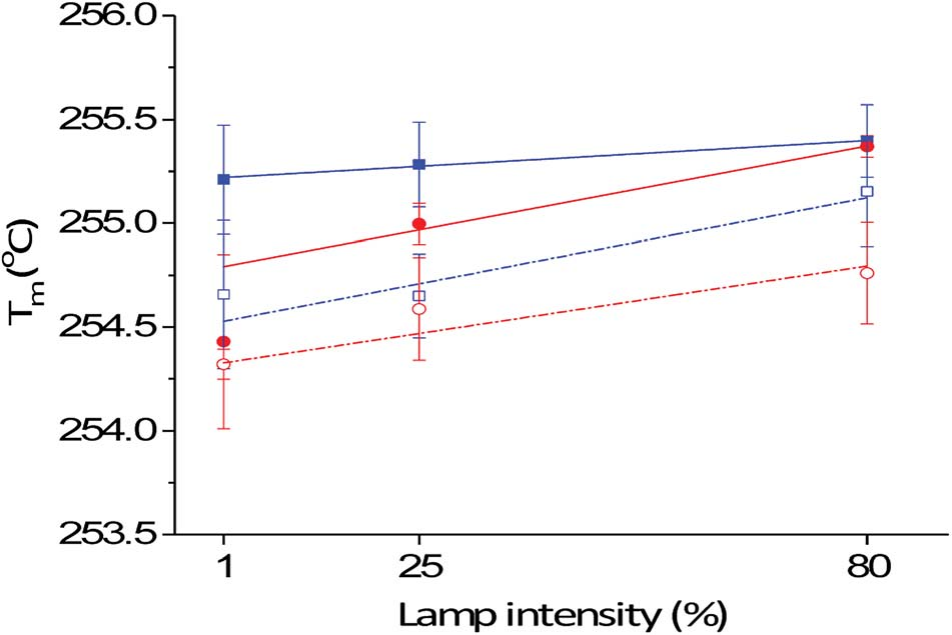
Comparison of melting peak temperatures *T*_m_ for 10-pass prints of printed samples transported at belt speed of 50 mm s^−1^ (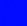) and 300 mm s^−1^ (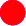) and washed samples transported at 50 mm s^−1^ (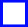) and 300 mm s^−1^ (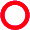). Trend lines indicate the linear fit of *T*_m_ as function of lamp intensity.

Thermal analysis of the interface between the printed photochromic ink and the PET fibre was measured by DSC. The cross-linked UV-ink gives no endothermic behaviour, however, it behaves as heat insulation layer, which affects the heat flow into PET during DSC measurement. The DSC measurement therefore gives indirect information on the polymer crosslinking density of the photochromic ink by measuring the melting temperature (*T*_m_) of the PET fabric with UV-cured photochromic prints. For prints with increased ink deposition, or cured with higher intensity, *i.e.* higher lamp intensity and/or lower belt speed, the ink has a higher crosslinking density. Hence, it forms a thicker or more distinct insulation layer on the PET fabric surface. This in return increases the *T*_m_ and differences in the melting peak temperature, *i.e.* Δ*T*_m_ of the PET fabric with photochromic prints on its surface compared to the untreated PET fabric. Inkjet-printed samples with different printing passes cured at a belt speed of 50 mm s^−1^ and 80% of the maximum lamp power (Fig. 3), showed a linear increase of *T*_m_ from 252.8 °C for 1 pass to 255.4 °C for ten passes. Prints with more amount of cross-linked polymer deviate more from the *T*_m_ of 252.9 °C of untreated PET fabric as they provide progressively more insulation. Furthermore, the prints showed a more significant shift of *T*_m_ with higher curing intensity (in combination with lamp intensity and belt speed). As shown in Fig. 4, an increase of lamp intensity, 1%, 25% to 80%, increases the *T*_m_ of the photochromic prints on PET. The prints produced at 300 mm s^−1^ showed a progressive increment of *T*_m_ with 254.4 °C, 255.0 °C and 255.4 °C, *i.e.* Δ*T*_m_ of 1.5 °C, 2.1 °C and 2.5 °C. A similarly increasing trend of *T*_m_ was detected for prints produced at 50 mm s^−1^ with various curing intensities but with less significant variation in Δ*T*_m_, *i.e.* 2.3 °C, 2.4 °C and 2.5 °C respectively. The saturation of *T*_m_ in prints produced at 50 mm s^−1^ indicates that the increase of curing power cannot further increase the crosslinking density in the UV-curable ink. The inkjet-printed photochromic material accounts for *ca.* 10 wt% of the measured DSC sample. To ensure representative measurements, the *T*_m_ and the standard deviation were taken from three independently measured samples. The largest standard deviation in DSC measurements is ±0.5 °C, which is in general smaller than presented in Table 1. Furthermore, an indium sample was measured three times with a *T*_m_ of 156.9 ± 0.05 °C, which is comparable with literature, where indium is reported with a *T*_m_ of 156.6 °C. Moreover, in order to exclude the effect of UV-radiation on *T*_m_ of the PET fabric, the *T*_m_ of UV-cured PET fabric only, *i.e.* non-printed, was measured by DSC with 254.0 ± 0.1 °C. In this case, the detected variation of Δ*T*_m_ (*T*_m_ of measured samples subtracted by 254.0 °C, Fig. 3(a)) which is reduced to −1 to 1.5 °C, however, is still more significant than the measurement error. It can be concluded that the detected variation of Δ*T*_m_ is small in DSC in general, but as it shows systematic variation and higher significance than the measurement error, it supports the validity of the experimental setup.

**Table 1:** Melting peak temperatures *T*_m_ of 10-pass prints at printed and washed condition for all curing setting combinations. Δ*T*_m_ denotes the difference in *T*_m_ between the printed and non-printed samples. Δ*T*_washed_ denotes the difference in *T*_m_ between the printed and washed samples and is a measure of print durability as a result of ink-fibre adhesion

Belt speed (mm s^−1^)	Lamp intensity (%)	*T*_m_ (°C) printed	*T*_washed_ (°C) washed	Δ*T*_m_ (°C) *T*_m_ – UV-treated PET	Δ*T*_washed_ (°C)
Untreated PET		252.9 ± 0.2	—		
300	1	254.4 ± 0.5	254.3 ± 0.1	0.4	0.1
	25	255.0 ± 0.1	254.6 ± 0.3	1.0	0.4
	80	255.4 ± 0.1	254.8 ± 0.2	1.4	0.6
50	1	255.2 ± 0.3	254.7 ± 0.4	1.2	0.5
	25	255.3 ± 0.2	254.7 ± 0.2	1.3	0.6
	80	255.4 ± 0.2	255.2 ± 0.3	1.4	0.2

Moreover, fitting and extrapolation of the data (Fig. S2 in ESI†) suggested that the *T*_m_ increased exponentially as a function of printing passes, which saturates at around 50 printed passes and a *T*_m_ of 257.1 °C. This indicates that measuring *T*_m_ with DSC can approximate the amount of cross-linked UV-ink at a certain curing condition on the PET fabric.

### Colour performance of the photochromic textile

#### Extended kinetic model for the colouration reaction

Colour measurements with the LCAM Photochrom 3 spectrophotometer have shown that the first-order kinetic model for the colouration reaction (eqn (2)) is only valid for photochromic prints with a high polymer crosslinking density, *i.e.* a belt speed of 50 mm s^−1^ and 80% lamp dosage (Fig. S3 in ESI†). For cured prints with a low polymer crosslinking density, hence lower *T*_m_, polymer crosslinking continues during colour measurements and a simultaneous secondary decay mechanism occurs. The trend of deviation from normal photochromic colouration behaviour is supported by DSC measurements. The lower the speed of the transportation belt and the higher the lamp intensity for curing, the higher is the *T*_m_ and hence the insulation effect of the cured ink on PET (Fig. 4). When using a conveyer belt speed of 50 mm s^−1^, the differences in polymer crosslinking density through curing at different radiation intensities, 1, 25 or 80%, become less crucial. At high belt speed, *i.e.* 300 mm s^−1^, increasing lamp power plays a decisive role in the crosslinking density that is achieved. Deviation from eqn (2) for the colouration reaction is in line with the degree of polymer crosslinking in the following order, starting from the curing settings with the highest deviation:

300 mm s^−1^/1% > 300 mm s^−1^/25% > 50 mm s^−1^/1% > 50 mm s^−1^/25% > 300 mm s^−1^/80% > 50 mm s^−1^/80%

**Fig. 5:**
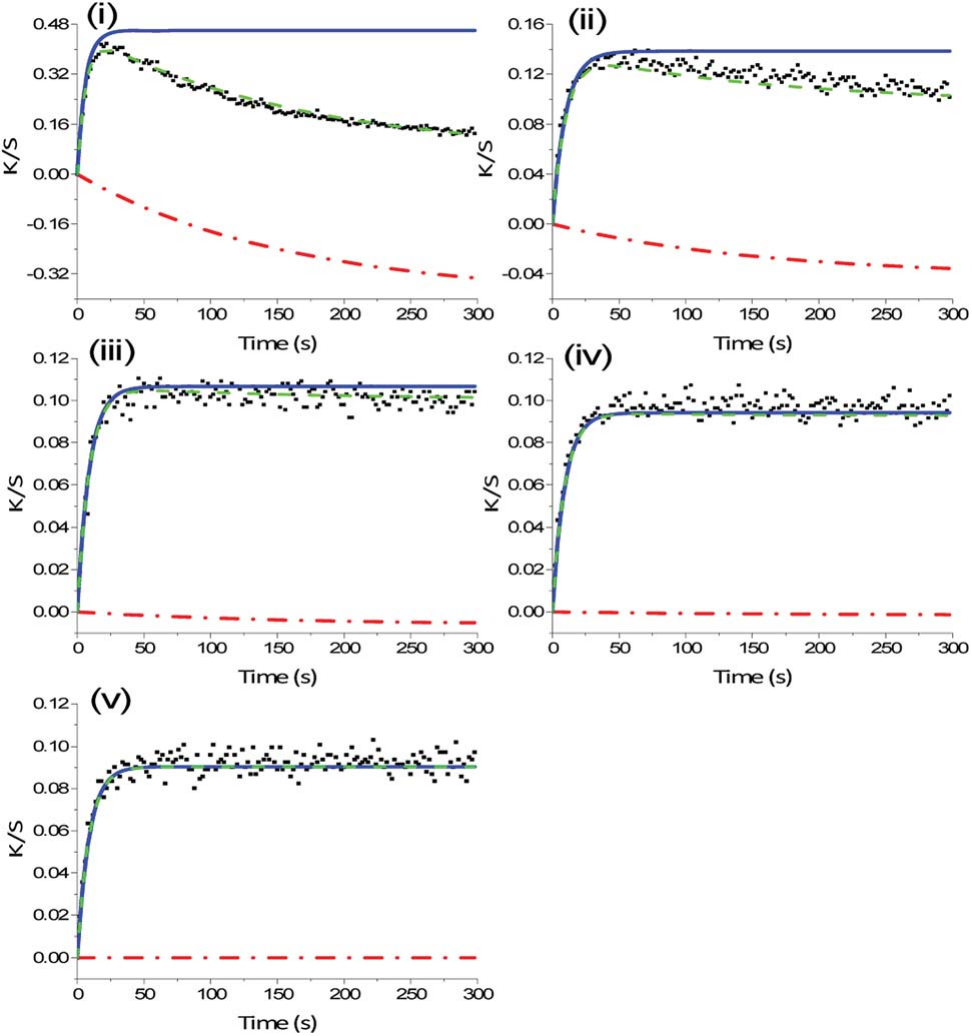
Extended model for reconstruction of photochromic colouration throughout colouration cycles 1–5 (i–v) of a print with 19 g m^−2^ deposited ink cured at belt speed of 300 mm s^−1^ and 1% lamp intensity. Measured *K*/*S* data (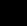) consists of reaction processes of decay (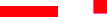) and colouration (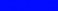) during colour measurement. The calculated sum of decay and colouration (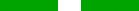) validates the model. For this print degradation becomes statistically ineffective in activation cycle 5 (v).

The combination of highest belt speed and lowest lamp intensity, 300 mm s^−1^ and 1%, has the highest deviation from the kinetic model in the first colouration cycle as a result of lowest polymer crosslinking density. This conforms to the colour data, which shows that UV-light exposure during colour measurement of samples with low crosslinking density decreased Δ*K*/*S*_colouration_ in the subsequent colouration cycles 2, 3, 4 and 5 (Fig. 5). This can be a result of continuous curing and simultaneous dye degradation. High UV-radiation improves the cure speed of the resin^32,33^ and longer irradiance increases the degree of polymer crosslinking.^21^ Meanwhile, photo-degradation of photochromic dyes is commonly observed under prolonged UV-irradiance.^34–36^

Therefore, for prints with lower polymer crosslinking density, we introduce a model that defines the kinetics of a decay mechanism as a result of continuous curing while colour measurement and reconstructs the actual colouration curve (Fig. 5). For ink systems, where UV-light is used to cure the photochromic prints with the aim to optimize curing conditions, the following extended kinetic model is proposed.3
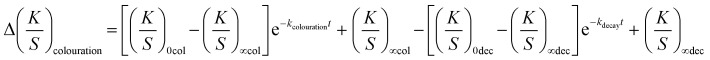
where *K*/*S*_0col_ is the initial colour value for the colouration reaction, *K*/*S*_∞col_ is the maximum colouration value, *K*/*S*_0dec_ is the initial value of the decay and *K*/*S*_∞dec_ is the final value of the degrading reaction for each activation cycle. Kinetic rate constants for the colouration reaction and decay are defined as *k*_colouration_ and *k*_decay_, respectively.

Under the boundary condition that the processes of colouration and decay start simultaneously and at Δ*K*/*S* = 0, which means that *K*/*S*_0col_ = 0 and *K*/*S*_0dec_ = 0, eqn (3) reduces to4
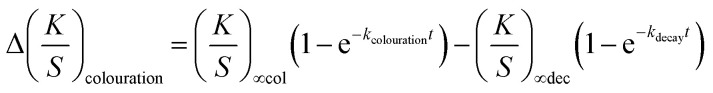


*Via* continuous UV-exposure over 1500 s it could be excluded that the decolouration reaction interferes with the observed decay mechanism during the colouration reaction (Fig. S5 in ESI†). Rate constants of the decay mechanism *k*_decay_ were fitted as exponential decay during UV-exposure, *i.e.* to the decrease in *K*/*S*_colouration_ throughout the five UV-exposure cycles during the colouration reaction, which add up to 1500 s.

The extended kinetic model, eqn (4), has been applied to the colour data of inkjet-printed samples with a deviation from eqn (2), *i.e. *R**^2^ < 0.95, to reconstruct the *K*/*S*_colouration_ values and *k*_colouration_, as well as to determine the extent of decay depending on the curing setting and applied amount of ink. An increasing amount of ink influences the secondary decay of the dye throughout colour measurement. It is a common trend for all curing settings that the lower the amount of applied ink, the higher is *k*_decay_. Prints cured at a belt speed of 50 mm s^−1^ and 1% lamp power show decreasing *k*_decay_ from 0.017 s^−1^ to 0.014 s^−1^ to 0.004 s^−1^ for 3, 5 and 10 printing passes, respectively. With increasing layer thickness oxygen diffusion is aggravated and the primarily affected surface area is proportionally reduced. As presented in Table 2, the secondary decay decreases with stronger curing conditions from *k*_decay_ = 0.006 s^−1^ for 300 mm s^−1^ and 1% to 0.002 s^−1^ for 50 mm s^−1^ and 25%. Also, variation in k_decay_ is higher when transported at 300 mm s^−1^. Hence, the decreasing trend of variation in *k*_decay_ and rates with increasing crosslinking density of prints directly correlates with the trend of deviation from eqn (2).

Colour yields Δ*K*/*S*_colouration_ and rate constants *k*_colouration_, *k*_decolouration_ and *k*_decay_ of 10-pass prints for all curing settings for printed and washed samples in activation cycle 1. Remained Δ*K*/*S* presents the percentage-wise residual colour yield Δ*K*/*S*_colouration_ after a washing process

**Table:** 

Belt speed (mm s^−1^)	Lamp intensity (%)	Printed			
		Δ*K*/*S*_colouration_	*k*_colouration_ (s^−1^)	*k*_decolouration_ (s^−1^)	*k*_decay_ (s^−1^)
300	1	0.467 ± 0.038	0.157 ± 0.018	0.011 ± 0.001	0.006 ± 0.003
	25	0.550 ± 0.049	0.128 ± 0.019	0.011 ± 0.001	0.004 ± 0.002
	80	0.434 ± 0.036	0.114 ± 0.018	0.010 ± 0.000	0.004 ± 0.002
50	1	0.376 ± 0.032	0.145 ± 0.019	0.010 ± 0.001	0.004 ± 0.001
	25	0.370 ± 0.012	0.123 ± 0.015	0.010 ± 0.000	0.002 ± 0.001
	80	0.278 ± 0.025	0.070 ± 0.008	0.008 ± 0.001	—

**Table:** 

Belt speed (mm s ^-1^)	Lamp intensity (%)	Washed			Remained Δ*K*/*S* (%)
		Δ*K*/*S*_colouration_	*k*_colouration_ (s^−1^)	*k*_decolouration_ (s^−1^)	
300	1	0.253 ± 0.028	0.109 ± 0.014	0.010 ± 0.001	54.1
	25	0.266 ± 0.007	0.086 ± 0.014	0.010 ± 0.001	48.3
	80	0.213 ± 0.020	0.066 ± 0.013	0.009 ± 0.001	49.0
50	1	0.206 ± 0.034	0.077 ± 0.020	0.009 ± 0.001	54.8
	25	0.185 ± 0.016	0.061 ± 0.011	0.009 ± 0.001	50.1
	80	0.154 ± 0.024	0.047 ± 0.006	0.007 ± 0.001	55.5

The intensity of the light source used for exposure in the LCAM Photochrom 3 spectrophotometer with 8 mW cm^−2^ at sample position is 750-times lower than the UV-LED lamp intensity, *i.e.* approx. 10-times lower than 1% intensity of the curing lamp. Also, exposure of the prints to UV-light stretches over 300 s during colour measurement instead of approx. 1 s during the curing process. Both low irradiation intensity and long exposure time of the photochromic dye in a liquid resin cause rapid degradation as a result of photo-oxidation. A partially liquid resin has a lower viscosity, which facilitates oxygen diffusion.^33^ It can be assumed that dye degradation as a result of photo-oxidation occurs in the soft component of the resin, which is more prominent compared to when the dye is fixed in a solid matrix. However, cut-off effects of the dye-carrying medium, *i.e.* shift in reflectance properties as a result of changed degree of cure and media thickness have also been investigated.^10,37^ We therefore consider the observed decrease a secondary decay, which obviously reduces Δ*K*/*S* and colour reaction kinetics.

#### Effect of amount of ink deposition on the colouration reaction

It was found that the achieved colour intensity Δ*K*/*S*_colouration_ of printed and washed samples was linear towards the deposited amount of photochromic ink irrespective of curing conditions (Fig. 7), which agrees with the linearly increasing *T*_m_ as function of ink amount. Stronger colour yields with increasing ink amount are visible by eye for both printed and washed samples as can be seen in Fig. 1(c). According to Viková *et al.*^13^ and Periyasamy *et al.*,^38^ deeper shades are expected with increased layer thickness as a result of lowered surface-bulk ratio and hence, less colourless reflection. Aldib^15^ also observed the trend of a continuous increase in colour yield from 1 to 10 inkjet printing passes of the same dye type but for solvent-based ink. This indicates that the crosslinking density and the adhesion of the UV-resin to the textile surface are not affected by the amount of ink deposition.

**Fig. 6:**
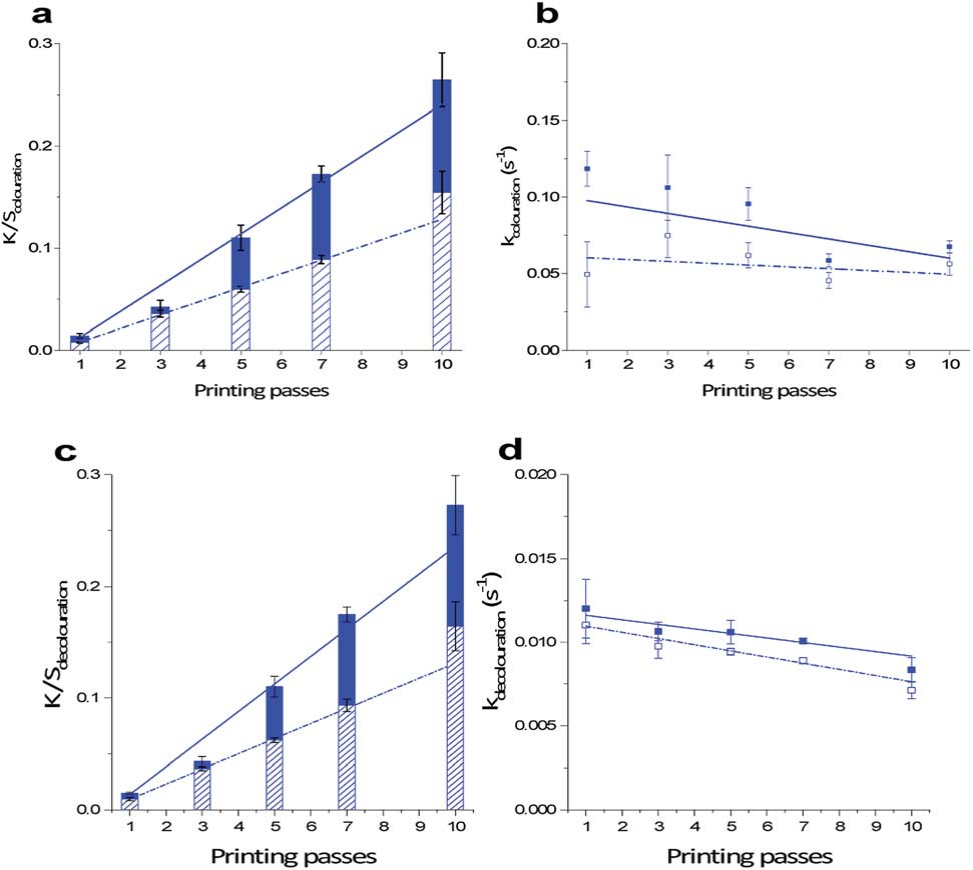
Trend lines indicate (a) linear increase of Δ*K*/*S*_colouration_, (b) successive decrease in *k*_colouration_, (c) linear increase of Δ*K*/*S*_decolouration_ and (d) successive decrease in *k*_decolouration_ as function of printing passes from 1 to 10 cured at a belt speed of 50 mm s^−1^ and 80% of lamp intensity printed (■) and washed (□).

The amount of printed photochromic ink on PET influences the kinetics of the colouration reaction. Similar to the trend in decrease in *k*_decay_, an increasing amount of applied material lowers *k*_colouration_ as seen in Fig. 6(b) for prints cured at a belt speed of 50 mm s^−1^ and 80% of lamp intensity. A decrease in *k*_colouration_ as function of printing passes can be explained by weakened activation of the photochromic dye with increased layer thickness.^10^ Washing has a significant effect on the colouration behaviour of the photochromic prints. Both Δ*K*/*S*_colouration_ and *k*_colouration_ significantly decrease as a result of a washing cycle. The reduced colour yield Δ*K*/*S*_colouration_ approximates 40% of the initial value for all printing passes from 1 to 10. With a decrease of approx. 20% at 10 passes compared to 40% at 1 pass, *k*_colouration_ is less affected with increasing layer thickness.

#### Effect of crosslinking density on the colouration reaction

**Fig. 7:**
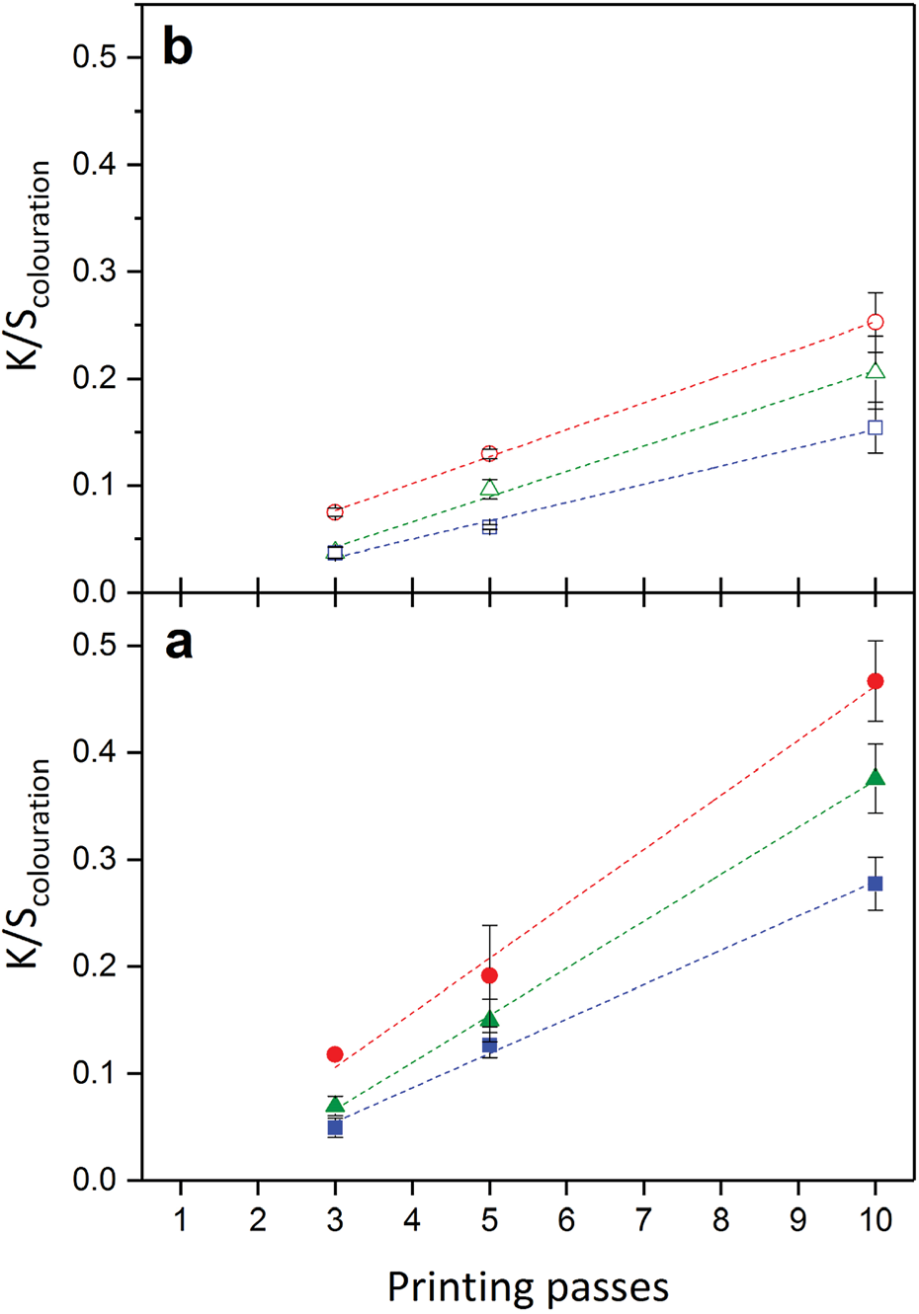
Linear increase of Δ*K*/*S*_colouration_ as a function of printing passes for low, intermediate and strong curing conditions, *i.e.* 300 mm s^−1^ + 1% (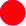,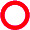), 50 mm s^−1^ + 1% (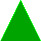,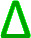) and 50 mm s^−1^ + 80% (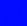,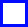), respectively after printing (a) and washing (b).

The production parameters belt speed and lamp intensity have a significant effect on Δ*K*/*S*_colouration_ and rate constants *k*_colouration_ and *k*_decay_. In Fig. 7, curing parameters belt speed and lamp intensity, 300 mm s^−1^ + 1%, 50 mm s^−1^ + 1% and 50 mm s^−1^ + 80% represent settings, which result in low, intermediate and high crosslinking density, respectively. Prints with high crosslinking density have experienced strongest curing, which increases the rigidity of the UV-resin but may also cause dye degradation. As a result, Δ*K*/*S*_colouration_ is lowered. As seen in Table 2, highest Δ*K*/*S*_colouration_ are achieved with curing at belt speed of 300 mm s^−1^ and low lamp intensity, *i.e. K*/*S*_colouration_ = 0.47 with 1% and *K*/*S*_colouration_ = 0.55 with 25%.

A high crosslinking density provides good ink-substrate adhesion, which is seen in high remaining Δ*K*/*S*_colouration_ values after washing. The remaining Δ*K*/*S* of 10-pass prints after a washing process is highest for strongest curing conditions, *i.e.* 55.5% for 50 mm s^−1^ and 80%. Washing decreases Δ*K*/*S*_colouration_ significantly for all curing conditions, with maximum remaining 55.5% to minimum 48.3% for 10-pass prints. It can be concluded that stronger curing conditions result in low absolute Δ*K*/*S*_colouration_ and low *k*_colouration_ and *k*_decolouration_. A low polymer crosslinking density of the carrier matrix exhibits a higher amount of soft component in the photopolymer, which enables more flexibility and free volume for faster switching of the photochromic dye molecule.^24,27^ A comparison of colour intensities and colouration rates between the six combinations of curing settings reveals that the setting with the lowest curing intensity, *i.e.* belt speed of 300 mm s^−1^ and lamp intensity of 1%, is the most favourable. High Δ*K*/*S* and fast kinetic switching with *k*_colouration_ of 0.157 s^−1^ after printing and 0.109 s^−1^ after washing between the uncoloured and coloured state of the textile UV-sensor is achieved. A higher cross-linked resin results in lowered rates of colouration with *i.e. k*_colouration_ of 0.070 s^−1^ before and 0.047 s^−1^ after washing for 10-pass prints cured at 50 mm s^−1^ and 80%.

Viková^10^ applied naphthopyran dyes on textiles in a screen-printing paste and achieved colouration rates of 0.09 s^−1^ and 0.25 s^−1^ with a concentration of 0.25 wt% at lamp intensity of 800 μW cm^−2^. The same dyes applied with a concentration of 1 wt% in a spin dope, which was melt-blown to a non-woven achieved colouration rates between 0.01 s^−1^ and 0.03 s^−1^.^10^ With respect to different concentrations and activation powers it can be assumed that similar dye types to Reversacol Ruby Red applied *via* a UV-cured matrix obtain *k*_colouration_, which are higher than in a fibre structure but lower than in a water-based and thermally cured acrylate paste.

#### Effect of amount of ink deposition on the decolouration reaction

The decolouration reaction as shown in Fig. 2 of naphthopyran dye Ruby Red is a thermal reaction, which takes place in the dark and therewith is independent from the colouration reaction.^27,39^ Decolouration yields Δ*K*/*S*_decolouration_, which correspond to Δ*K*/*S*_colouration_, and independent decolouration rates *k*_decolouration_, are calculated using eqn (2). A significant difference is seen for Δ*K*/*S*_decolouration_ after printing and after a washing cycle. The same behaviour as for Δ*K*/*S*_colouration_ is seen for decolouration intensities. As seen in Fig. 6(c), Δ*K*/*S*_decolouration_ increase linearly as function of printing passes and exhibit approx. 60% of the initial yields after washing. It is noted that for small amounts of printed ink, *i.e.* 1 and 3 printing passes, only 35% and 15% of the initial Δ*K*/*S*_decolouration_ is lost during the washing process, respectively.

With an increasing amount of printed ink, *k*_decolouration_ to revert the photochromic dye back to its uncoloured state are successively lowered by *ca.* 30% when cured with low speed and high lamp intensity, *i.e.* 50 mm s^−1^ and 80%. *k*_decolouration_ decreases from 0.012 s^−1^ for 1-pass prints to 0.008 s^−1^ for 10-pass prints. A washing process has a significant effect and lowers the decolouration rates by 10–15% for all printing passes (Fig. 6(d)). Same as for *k*_colouration_, *k*_decolouration_ is influenced by the thickness of the applied ink on the textile surface. An increase in layer thickness decreases the reaction rate.

#### Effect of crosslinking density on the decolouration reaction

The crosslinking density has a statistically significant effect on Δ*K*/*S*_decolouration_ irrespective of the amount of printed ink, *i.e.* 1 to 10 passes, on PET before and after washing. The higher the speed of the conveyer belt, the higher is Δ*K*/*S*_decolouration_. It is also valid, that the lower the UV-dosage during curing, the higher is the expected Δ*K*/*S*. Decolouration rates are most affected by strong curing conditions, that means 50 mm s^−1^ belt speed and 80% lamp intensity. *k*_decolouration_ follows the same trend as Δ*K*/*S*_decolouration_ values as a result of change in belt speed and lamp power, *i.e.* crosslinking density. The lower the crosslinking density, the least rigid is the UV-resin and hence the higher is *k*_decolouration_ of the photochromic dye to its uncoloured state. For 10-pass prints transported with a belt speed of 300 mm s^−1^ and cured with a lamp intensity of 1% *k*_decolouration_ is nearly unchanged with a rate of 0.011 s^−1^ after printing and 0.010 s^−1^ after washing as listed in Table 2. Curing at this condition allows the production of photochromic prints with an expected Δ*K*/*S* = 0.25 after washing and switching on of the photochromic effect at a rate of 0.11 s^−1^. Impact on the change in *k*_colouration_ and *k*_decolouration_ achieved by differences in conveyer belt speeds and lamp intensities during the curing process is less compared to other means of polymer architecture. Ercole *et al.*^25^ and Malic *et al.*^26^ studied that mid placement of the photochromic dye in homo-polymers can optimize photochromic kinetics. A similar effect can be achieved by tuning the polymer length and rigidity of the polymer matrix. Whereas, *via* polymer conjugation and, of course with respect to the generic switching speed of the chosen dye molecule, decolouration rates of a naphthopyran dye can be doubled to increased up to a five-fold from 0.01 to 0.05 s^−1^,^26^ by variation of fabrication parameters during curing approx. 1.4-times faster *k*_decolouration_ can be obtained.

### Durability and adhesion of the photochromic ink

#### Effect of deposited ink amount and curing settings on the print durability

Washing, which is an essential feature in textile applications, gives information on the durability and adhesion of a functional treatment on the textile surface. Here, the difference in *T*_m_ of the photochromic prints can be applied as a measure of durability in terms of adhesion of the ink on the PET surface. The durability of photochromic prints is assessed by measurement of *T*_m_ in DSC after a standard washing cycle (ISO 6330), *i.e.* the difference between *T*_m_ and the melting peak temperature after washing (*T*_washed_), *i.e.* Δ*T*_washed_. The more cross-linked UV-ink is left on the PET surface after a washing cycle, the more durable is the functional surface, and hence, the smaller the Δ*T*_washed_. The effect of washing (Δ*T*_washed_) gives indirect information on the durability of the cross-linked photochromic ink and its adhesion to the surface of PET.

Washed samples exhibit a statistically significantly lowered melting temperature *T*_washed_ regardless of the curing condition, as shown in Fig. 4. The decreased *T*_washed_, as listed in Table 1, is a measure for durability in terms of adhesion of the cured photochromic print to the PET fabric surface. While the Δ*T*_washed_ for prints cured at 300 mm s^−1^ with 1% lamp power remains nearly unchanged with a decrease of 0.1 °C, for prints transported at a belt speed of 50 mm s^−1^, the trend of the *T*_m_ as a function of lamp intensity changes after washing. Photochromic prints that underwent strongest curing at 80% of the maximum lamp power had almost no change in Δ*T*_washed_ (0.2 °C within the measurement error). Prints that have been cured at both 25% and 1% lamp intensity have a *T*_washed_ of 254.7 °C. This gives a Δ*T*_washed_ of 0.6 °C and 0.5 °C for lamp powers 25% and 1%, respectively. The result for the samples processed with 50 mm s^−1^ suggested that higher lamp power could increase the crosslinking density in the printed UV-ink.

Furthermore, the combination of *T*_washed_ and Δ*T*_washed_ assesses the durability of the functional layer with different crosslinking densities, *e.g.*, the combination of high *T*_washed_ and small Δ*T*_washed_ gives an indication for a durable photochromic layer with high crosslinking density in the prints. Prints cured at 50 mm s^−1^ and 80% have the highest *T*_washed_ of 255.2 °C and a Δ*T*_washed_ of 0.2 °C. It implies that a higher polymer crosslinking density is achieved as the print creates a strong insulation layer on the PET surface. Prints cured with the lowest curing intensity, *i.e.* 300 mm s^−1^ and 1%, exhibit a lower polymer crosslinking density, but are durable and flexible as the *T*_washed_ is the lowest with 254.3 °C (with Δ*T*_washed_ of 0.1 °C).

As shown in Fig. 3(b), washing has an effect on the inkjet-printed samples cured at a belt speed of 50 mm s^−1^ and 80% lamp intensity from 1 to 10 printing passes. The slope of the linear fit of *T*_washed_ as a function of printing passes is slightly lower than the fit of *T*_m_. However, changes in *T*_washed_ from 253.8 °C to 253.5 °C (Δ*T*_washed_ of 0.3 °C) for 3 passes and from 255.4 °C to 255.2 °C (Δ*T*_washed_ of 0.2 °C) for 10 passes are small (Fig. 3(b)).

The durability of the functional layer (Δ*T*_washed_) related to process parameters can be confirmed by the difference in Δ*K*/*S* from colour measurements after washing. The samples produced at 300 mm s^−1^ and 1% exhibit 54.1% of the initial colour yield, whereas the sample produced at 50 mm s^−1^ and 80% preserves 55.5% of its initial colour after one wash. Both curing conditions show decent durability assuming that the colour performance is in linear relationship with the amount of UV-ink printed (remained) on the PET fabric surface. Furthermore, the weight of printed and washed functional layers is complimentary to DSC and colour measurements (ESI†). As shown in Fig. S5,† the samples produced with various amounts of ink deposition cured at 50 mm s^−1^ and 80% were weighed after printing and washing. The weight of the functional layer, with the exception of 1-pass prints (measured weight loss of 100%), decreased by an average of 23 ± 12%. The weight decrease is due to a loss of the photochromic print during washing, which correlates with the decreasing trends in *T*_m_ and Δ*K*/*S*.

The result confirms that a belt speed of 50 mm s^−1^ and lamp intensity of 80% are valid process parameters to obtain a durable functional layer with high crosslinking density in prints. In summary, both a belt speed of 300 mm s^−1^ and lamp intensity of 1% and a belt speed of 50 mm s^−1^ and lamp intensity of 80% can produce a durable functional layer but the latter results in a higher crosslinking density in prints. The *T*_washed_ of the photochromic prints on PET after one washing cycle reaches a mean value of 254.7 °C for all curing settings. Here, statistical analysis shows that differences in belt speed and lamp intensity are insignificant in regards to *T*_m_ and *T*_washed_. Despite an increasing trend in crosslinking density with lower belt speeds and higher lamp powers, it can be assumed that stronger curing intensities than 300 mm s^−1^ and 1% are not more beneficial for the durability of the print.

## Conclusions

The colour performance of a novel UV-curable and UV-responsive smart textile is optimized using the combination of two challenging functions of electromagnetic radiation, where high energy UV-rays both cure and activate UV-sensitive naphthopyran dye Ruby Red. Tuning of the crosslinking density of the photochromic ink and hence the dye kinetics using fabrication parameters is an important step towards the design of a textile UV-sensor. Irrespective of the deposited ink amount on the PET surface between 1 and 10 printing passes, curing with the highest belt speed of 300 mm s^−1^ combined with the lowest lamp intensity 1% of the pilot-scale inkjet printing system achieves high colour yields Δ*K*/*S* with 0.47 after printing and 0.25 after washing, fastest isomerization with *k*_colouration_ of 0.16 s^−1^ after printing and 0.11 s^−1^ after washing and good durability after washing with 54.1% of the initial Δ*K*/*S*, despite lowest polymer crosslinking density. The effect of the solid photopolymer resin compared to waterborne or solvent-based carriers for a photochromic dye, similar to Reversacol Ruby Red, applied on textiles results in slower switching rates between the coloured and uncoloured states, but faster than in mass-dyed and melt-spun fabrics. Our findings add to the general trend of enhancing switching rates between the different isomers of photochromic dyes, as well as the overall colour yield as a result of reduced rigidity of the polymer matrix. *Via* a new kinetic model, which takes a decay mechanism of the dye upon colour measurement into account, the colour performance can be predicted and tuned based on fabrication parameters. Beyond tuning of colour kinetics, the novel and industrially applicable approach of UV-LED curing of photochromic inkjet ink is favourable in regards to the flexibility, resource-efficiency and cost-effectiveness of the production process. Inkjet printing combined with UV-LED curing has large potential to trigger innovative products and to promote the small-batch production of functional high-end and smart textiles.

## Conflicts of interest

There are no conflicts to declare.

## Supplementary Material

RA-008-C8RA05856C-s001

RA-008-C8RA05856C-s002
