# Supplementary material for: Inkjet printing and UV-LED curing of photochromic dyes for functional and smart textile applications
Source: RSC Adv. 2018 Aug 8;8(50):28395–404. doi: 10.1039/c8ra05856c (PMC9084306; doi:10.1039/c8ra05856c)
Supplement: RA-008-C8RA05856C-s002 [file RA-008-C8RA05856C-s002.pdf]

Electronic Supplementary Material (ESI) for RSC Advances.
This journal is © The Royal Society of Chemistry 2018

Inkjet printing and UV-LED curing of photochromic dyes for functional and smart textile applications

Sina Seipel[1]*, Junchun Yu[1], Aravin P. Periyasamy[2], Martina Viková[2], Michal Vik[2] and Vincent A. Nierstrasz[1]

[1] Textile Materials Technology, Department of Textile Technology; Faculty of Textiles, Engineering and Business; University of Borås; 50190 Borås, Sweden.
[2] Department of Material Engineering; Faculty of Textile Engineering; Technical University of Liberec; 461 17 Liberec, Czech Republic.

E-Mails: junchun.yu@hb.se; vincent.nierstrasz@hb.se; martina.vikova@tul.cz; michal.vik@tul.cz; aravinprince@gmail.com

*Author to whom correspondence should be addressed:
E-Mail: sina.seipel@hb.se;
Tel.: +46-33-435 4191.

Supplementary information

S1. UV-Vis absorption spectra of ink components

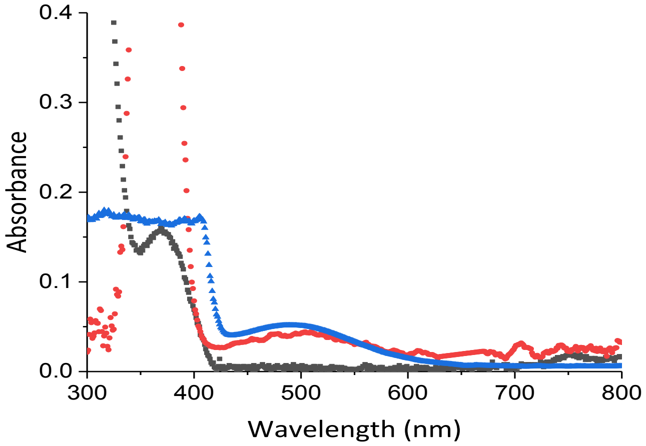

Figure S1. UV-Vis absorption spectra of 50 mg L^{-1} photo-initiator TPO-L in methanol (■) and 100 mg L^{-1} photochromic dye Ruby Red in UV-resin (▲) and 100 mg L^{-1} Ruby Red in ethyl acetate (●), respectively.

S2. Crosslinking density of the UV-curable ink

According to the best fit of data an expontential trend of the melting peak temperature T_m as funtion of printing passes is assumed with an extrapolated saturated T_m of 257.1 °C at 60 printing passes. However, in the practical range from 1 to 10 printing passes a linear fit is proposed.

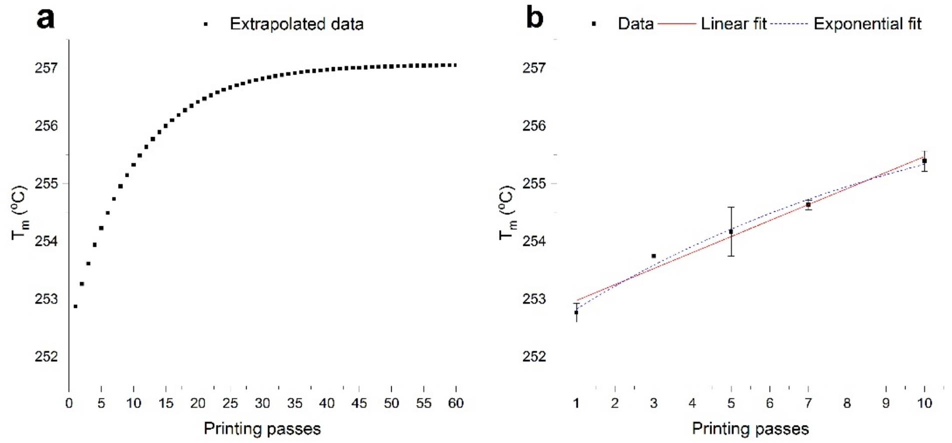

Figure S2. (a) Exponential extrapolation of measured data points for prints cured at 50 mm s^{-1} and 80% results in a T_m of 257.1 °C for 60 printing passes of ink. (b) Linear and exponential fit of measured T_m for 50 mm s^{-1} + 80% prints from 1 to 10 printing passes with R^2_{exp} of 0.978 and R^2_{lin} of 0.973.

S3. Development of the extended kinetic model

While the first activation cycle (a) during the colouration phase of a print cured at a belt speed of 50 mm s^{-1} and 1% lamp intensity deviated from Eqn. (2), the second colouration cycle (b) is described with acceptable fit using Eqn. (2) for photochromic colouration (Fig. S3). However, this results in a lowered $\Delta K/S_{colouration}$ due to a secondary decay mechanism while activation with UV-light during measurement.

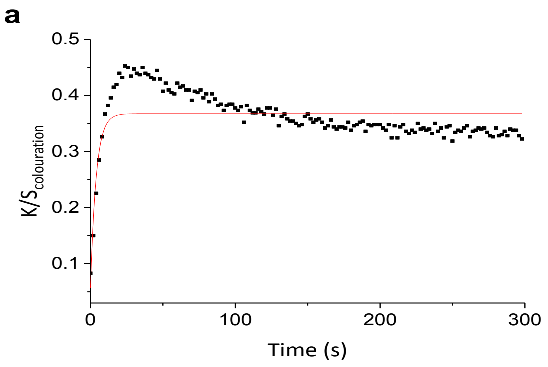

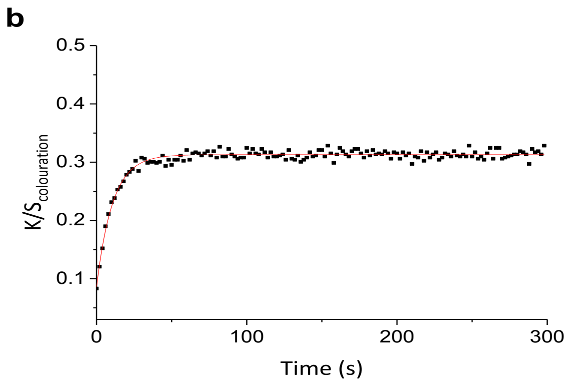

Figure S3. $\Delta K/S_{colouration}$ (■) and first order curve fitting (-) according to Eqn. (2) of a photochromic print with 19 g m^{-2} deposited ink, cured at belt speed of 50 mm s^{-1} and 1% lamp intensity in (a) cycle 1 with a fitting value $R^2 < 0.45$ and (b) cycle 2 with $R^2 > 0.95$.

Decay of photochromic prints with low crosslinking density is observed upon UV-exposure. As shown in the Figure S4, via continuous UV-exposure over 1500 s it could be excluded that the decolouration reaction interferes with the decay during the colouration reaction. Fitting of the decay curves and their rate constants k_{decay} during UV-exposure, i.e. during the colouration reaction is done based on the decrease in K/S throughout the five UV-exposure cycles as seen in the green data.

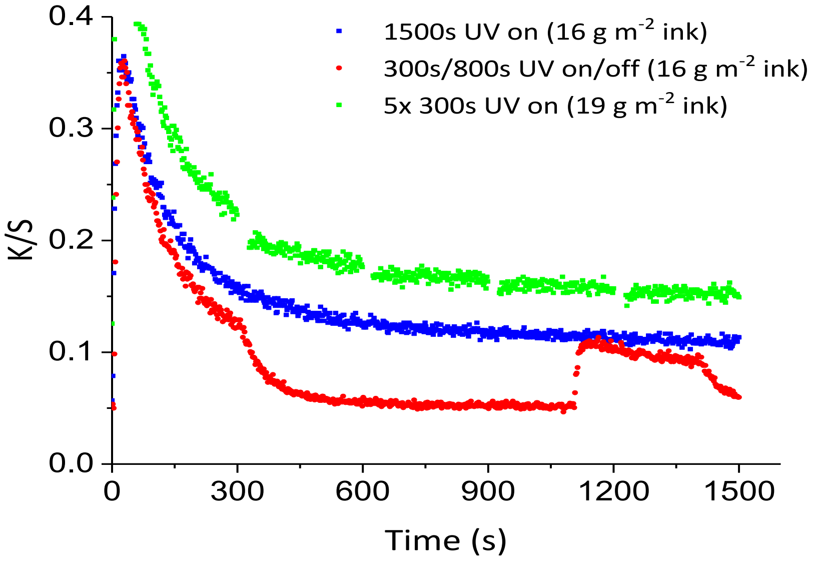

Figure S4. Decay curves during UV-exposure (and relaxation) of photochromic prints cured at 300 mm s^{-1} + 1% with different deposited ink amount during spectrophotometry with Photochrom 3. (■) 1500 s continuous UV-exposure of print with ink amount of 16 g m^{-2}, (●) Alternating 300s UV-exposure and 800 s relaxation of print with ink amount of 16 g m^{-2} and (▲) Five 300 s UV-exposure cycles of print with 19 g m^{-2} deposited ink.

S5. Weight difference of prints after washing

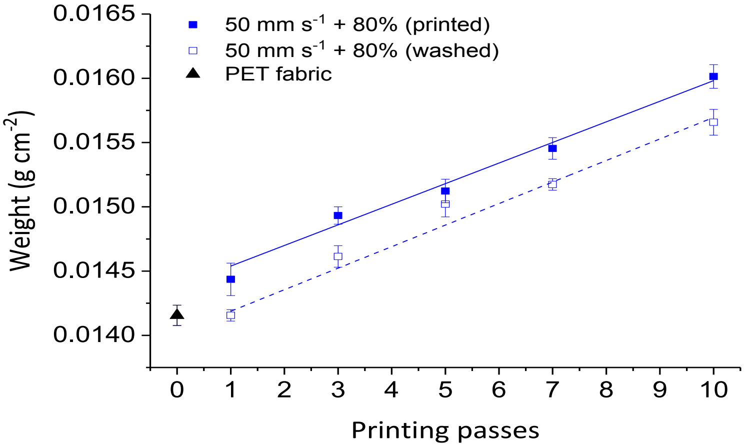

Figure S5. Weight difference of printed (■) and washed (□) samples cured at 50 mm s^{-1} belt speed and 80% lamp intensity compared to the untreated PET fabric (▲).
